# Innovative Approaches for the Treatment of Spinal Disorders: A Comprehensive Review

**DOI:** 10.26502/josm.511500190

**Published:** 2025-03-27

**Authors:** Edgmin Rostomian, Kevin Ghookas, Alexander Postajian, Kevin B Vartanian, Vedi Hatamian, Marcel P Fraix, Devendra K. Agrawal

**Affiliations:** Department of Translational Research, College of Osteopathic Medicine of the Pacific, Western University of Health Sciences, Pomona, California 91766 USA

**Keywords:** Accessibility challenges, Artificial disc replacement, Artificial intelligence, Complex deformities, Disc replacement, Minimally invasive surgery, Non-surgical treatment, Personalized medicine, Regenerative medicine, Spinal disorders, Wearable technologies

## Abstract

This comprehensive review explores the latest advancements in the management of spinal disorders, including minimally invasive surgical techniques, treatment of complex deformities, disc replacement technologies, and non-surgical approaches. The review highlights the potential of innovations such as robotic-assisted surgeries, regenerative medicine, and artificial intelligence to enhance precision, reduce recovery times, and improve patient outcomes. It also discusses the integration of wearable technologies and personalized medicine in tailoring treatments. Challenges such as high costs, accessibility issues, and limited long-term data are critically analyzed, alongside gaps in research, including a lack of diversity in study populations and insufficient economic evaluations. Future directions emphasize the need for multidisciplinary collaboration to develop durable, accessible, and personalized solutions to address the global burden of spinal disorders.

## 1. Overview of Spinal Disorders

The spine is a complex structure comprising numerous joints, ligaments, and muscles, playing a pivotal role in supporting body weight, enabling movement, and protecting the spinal cord. Disruptions to this intricate system, such as herniated discs, degenerative disc disease, and traumatic injuries, are leading causes of chronic pain and functional impairments globally [1]. According to a systematic analysis of the Global Burden of Disease Study, low back pain is among the leading contributors to disability-adjusted life years (DALYs) for musculoskeletal disorders globally, highlighting its significant societal impact [2]. In the United States, the economic toll of lower back pain, a sign of spinal disorders, exceeds billions annually due to healthcare costs, lost productivity, and disability compensation [3]. Low back pain is a significant contributor to musculoskeletal healthcare expenses in Europe as well, representing a major cause of economic burden through substantial healthcare utilization and lost productivity among working-age populations [4,5]. Moreover, disparities in access to spinal care contribute to heightened chronic disability in underserved regions due to limited diagnostic tools and therapeutic resources [6].

Emerging strategies aim to address these challenges. Innovations like minimally invasive surgery (MIS), regenerative treatments, and combined technologies such as artificial intelligence (AI) and wearable devices hold promise in revolutionizing spinal care. These advancements aim to improve clinical outcomes, reduce recovery times, and alleviate economic strains by providing cost-effective, efficient, and equitable solutions. This review explores these innovations, assessing their effectiveness, applications, and potential to reshape spinal disorder management while outlining opportunities for further progress in this critical field.

## 2. Advancements in Treatment

The field of spinal disorder treatment has experienced a transformative change, shifting away from traditional methods to innovative approaches that challenge established constraints. While conventional approaches, such as physical therapy, medication for pain relief, and traditional surgeries, have been historically effective, they often involve extended recovery times, substantial risks of complications, and variable outcomes [3]. In response to these challenges, the field has embraced minimally invasive techniques, regenerative therapies, and integrative technologies, which are redefining the landscape of spinal care.

Minimally invasive surgery has emerged as groundbreaking, utilizing innovations such as endoscopic discectomy, percutaneous vertebroplasty, and robotic-assisted spinal fusion to decrease tissue damage, speed up recovery, and lower procedural risks. Robotic-assisted systems have demonstrated significant improvements in pedicle screw placement accuracy, reducing the incidence of intraoperative complications such as bleeding and infection compared to traditional methods [8]. Studies on percutaneous vertebroplasty have reported rapid pain relief and improved functional mobility, particularly in patients with osteoporotic vertebral fractures [9,10].

Regenerative medicine approaches are redefining treatment paradigms, particularly for patients unsuitable for surgical interventions. Platelet-rich plasma (PRP) therapies have been shown to accelerate healing by targeting inflammatory processes and promoting tissue regeneration in degenerative disc disease [11]. Furthermore, mesenchymal stem cell therapies are emerging as promising alternatives, with experimental data indicating enhanced extracellular matrix production and intervertebral disc hydration in preclinical models [12–14].

The integration of advanced technologies is further challenging existing norms. Artificial intelligence (AI) aids in diagnostic accuracy and surgical planning, as demonstrated in studies where AI-based imaging systems achieved superior vertebral alignment predictions compared to traditional methods [15,16]. Additionally, innovations in spinal implants, including motion preserving prosthetics and 3D-printed devices, are reshaping the management of spinal pathologies. Studies have shown that motion-preserving prosthetics, such as artificial discs and dynamic stabilization systems, may reduce the risk of adjacent segment degeneration compared to traditional fusion techniques [17,18]. Moreover, 3D-printed implants, designed for better biocompatibility and load distribution, have demonstrated promising outcomes in preclinical and early clinical trials, supporting their potential to optimize spinal stability and reduce mechanical stress on adjacent segments [19,20].

Although these developments represent extraordinary progress, many challenges exist. High costs and steep learning curves associated with robotic systems pose barriers to their widespread adoption, particularly in community hospitals [21,22]. Similarly, regenerative therapies, while promising, are constrained by limited cost-effectiveness analysis and an absence of long-term safety data [23]. Further large-scale, multicenter studies are required to establish comprehensive guidelines and ensure equitable access to these transformative treatments.

## 3. Risk Factors

**Figure 1:**
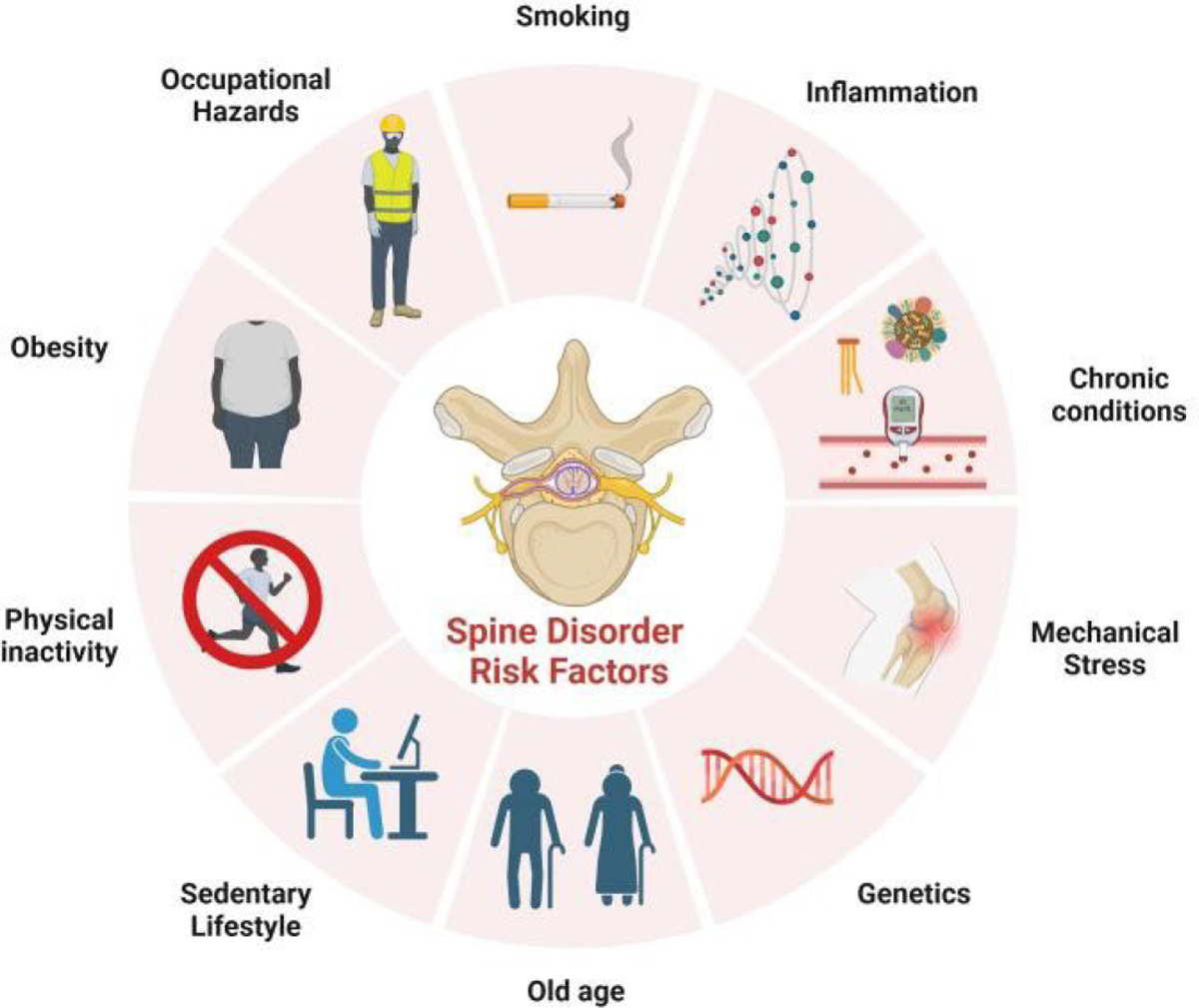
Risk factors associated with spine disorders.

The onset of spinal disorders is the result of a complex interplay of internal and external risk factors as seen in Figure 1. Aging is a prevalent factor that leads to degenerative changes in the spine, such as wear and tear of intervertebral discs, reduced bone strength, and thickening of ligaments, which increases the likelihood of developing issues like spinal stenosis and degenerative disc disease. Studies have shown that the prevalence of lumbar spinal stenosis significantly rises in individuals aged 60 and older, with systematic reviews estimating about 14% of elderly populations display clinical or radiological signs of stenosis [24]. Similarly, research highlights the growing impact of degenerative disc diseases in elderly populations, emphasizing the pressing need for targeted interventions [25].

Additionally, occupational factors exacerbate risks, with physically demanding jobs such as construction showing a significantly higher incidence of lumbar disc herniation and chronic back pain (Figure 1). Construction workers have a significantly higher risk of developing lumbar disc herniation compared to sedentary occupations, with ergonomic interventions demonstrating potential to mitigate this risk by addressing physical demands and improving workplace safety [26,27]. As life expectancy continues to climb around the world, the societal impact of age-related diseases is likely to increase, amplifying the need for focused interventions.

Lifestyle habits, characterized by a lack of physical activity, obesity, and smoking, intensify spinal disorder susceptibility. Obesity has been causally associated with intervertebral disc degeneration, with studies indicating that a higher body mass index significantly increases the risk of lumbar disc degeneration due to mechanical stress and systemic inflammation [28,29]. Smoking has a dose-dependent relationship with intervertebral disc degeneration, impairing blood flow to spinal structures and exacerbating inflammatory processes, contributing to progressive disc deterioration [30]. Conversely, physical activity strengthens paraspinal muscles and improves musculoskeletal health, reducing the likelihood of spinal instability and degenerative conditions [31].

Genetics adds another layer of complexity, as inherited traits can affect conditions such as scoliosis and disc degeneration. Heritability studies indicate a substantial genetic contribution to lumbar disc degeneration, with research highlighting the role of inherited factors and genetic predisposition in its development [32]. Understanding this genetic connection offers the potential for tailored treatment options, highlighting the importance of comprehensive prevention strategies to reduce the incidence of spinal disorders.

## 4. Etiology

The causes of spinal disorders are complex and arise from various primary factors that distinctly influence their development and progression. Degenerative changes due to agerelated alterations in spinal structure are at the forefront. As time passes, intervertebral discs dry out and become less elastic, leading to conditions such as degenerative disc disease, instability, or spinal stenosis. Recent studies have confirmed that such changes, compounded by osteoarthritis and facet joint degeneration, significantly contribute to dysfunction in aging populations [33]. Additionally, lumbar spinal stenosis is frequently observed in aging populations, with notable clinical symptoms leading to substantial healthcare utilization and societal impact, as highlighted in recent analyses [34].

Trauma stands out as another significant factor, including sudden injuries such as fractures, dislocations, or ligament tears caused by high-energy events like car accidents or falls. If these injuries are not addressed, they can lead to long-term disabilities and neurologic issues. More subtle repetitive stress injuries, often linked to certain occupations, can gradually lead to chronic spinal problems. Workers in physically demanding roles exhibit a significantly higher prevalence of degenerative musculoskeletal changes, as highlighted by systematic reviews. This highlights the importance of occupational health interventions to mitigate risks and enhance workplace safety [35,36].

Inflammatory conditions, such as ankylosing spondylitis and rheumatoid arthritis, significantly contribute to spinal disorders. Ankylosing spondylitis predominantly affects the sacroiliac joints and axial skeleton, resulting in progressive rigidity and deformity due to chronic inflammation and abnormal bone formation [37]. Rheumatoid arthritis, on the other hand, frequently involves the cervical spine, leading to atlantoaxial instability or subluxation and potential neurological deficits if untreated [38]. Both conditions arise from autoimmune mechanisms that drive inflammation, cartilage destruction, and bone erosion. Early intervention with disease-modifying antirheumatic drugs and biologics has improved patient outcomes, but delayed treatment increases the risk of severe complications [39].

Neoplastic involvement, which can be either primary (e.g., chordomas) or metastatic (e.g. from breast to lung cancer), adds to the complexity, often leading to fractures or spinal cord compression that requires a multidisciplinary approach. Spinal metastases represent a significant proportion of osseous metastatic cases, frequently leading to structural instability and neurological complications, underscoring their critical importance in oncological care [40]. This complicated etiology elucidates the need for targeted therapeutic strategies to tackle the multifaceted cause of spinal disorders.

## 5. Incidence

The occurrence of spinal disorders has increased significantly around the world, driven by changes in demographics, lifestyle patterns, and inequities in healthcare accessibility. Low back pain is one of the most prevalent and disabling conditions globally, significantly associated with aging populations. Conditions like osteoarthritis, disc degeneration, and spinal stenosis increase in prevalence with age, and projections indicate that by 2050, the global population aged 60 and older will nearly double, intensifying healthcare challenges [41,42].

Regional differences reveal the impact of occupational hazards and lifestyle choices. Occupations with poor ergonomic practices, such as manual labor, are strongly associated with musculoskeletal disorders. Preventive ergonomic interventions have been shown to significantly reduce these risks [43]. Sedentary lifestyles and rising obesity rates contribute to increased spinal strain and elevate the risk of degenerative conditions such as lumbar disc degeneration and osteoarthritis [44,45].

Economic disparities also impact this epidemiological trend. Limited access to healthcare in low-income regions often results in advanced-stage diagnoses, while high-income countries report higher utilization of advanced imaging and surgical interventions [46]. Addressing these global disparities will require targeted strategies to reduce the burden of spinal disorders effectively.

## 6. Underlying Pathogenesis

### 6.1 Mechanical Instability

Mechanical instability of the spine arises from structural disruptions that compromise its ability to maintain proper alignment and distribute loads during physiological activities. Disc degeneration, a major factor, reduces shock absorption and intervertebral spacing as hydration and elasticity decrease over time. This leads to increased mechanical stress, which exacerbates further degeneration and instability [47]. Herniated or bulging discs, commonly associated with nerve root compression, are significant contributors to radicular pain and motor deficits. Studies highlight the prevalence of instability and functional impairments in such cases, necessitating early diagnosis and intervention [48,49].

Another key contributor to mechanical instability in spinal disorders is the dysfunction of spinal musculature and ligamentous structures. Paraspinal muscle weakness, particularly in the multifidus and erector spinae, reduces the ability of spine to maintain proper alignment and resist abnormal movement, significantly contributing to instability. Research highlights the importance of muscle endurance and recruitment in maintaining segmental stability and mitigating degenerative changes [50]. Ligamentous laxity, often resulting from repetitive stress or degenerative conditions, exacerbates this instability by allowing excessive motion at intervertebral joints, which further stresses surrounding structures and accelerates degeneration [51]. Together, these impairments create a vicious cycle of instability and degeneration, underscoring the need for targeted rehabilitative and, in severe cases, surgical interventions.

Vertebral misalignments, such as spondylolisthesis and scoliosis, further disrupt the biomechanical equilibrium of the spine. Spondylolisthesis, characterized by the forward slippage of a vertebra, creates excessive stress on adjacent segments and accelerates degeneration [52]. Scoliosis leads to uneven spinal loading, which contributes to progressive degeneration and chronic pain. Clinical studies show that increasing Cobb angles are associated with mechanical instability, particularly in cases of significant spinal deformity, exacerbating functional impairments [53,54]. These degenerative, inflammatory, and structural factors collectively create biomechanical instability, necessitating targeted interventions to restore function and alleviate symptoms.

### 6.2 Neurologic Impact

The neurological effects of spinal disorders are significant, resulting in pain, sensory changes, and functional impairment, stemming from the interaction of nerve compression and inflammatory responses. Structural issues like herniated discs or osteophytes intrude upon spinal nerve roots or the spinal cord itself, hindering neural communication and triggering symptoms such as radicular pain, tingling, and muscle weakness [55]. Lumbar disc herniation commonly compresses the exiting nerve root within the neural foramen, leading to sciatica characterized by radiating leg pain. Displaced disc material is a significant cause of nerve root compression associated with sciatica [56].

In addition, inflammation worsens neural dysfunction. Disc injury and degeneration activate proinflammatory mediators, such as tumor necrosis factor-alpha (TNF-α) and interleukin-1 beta (IL-1β), which sensitize nociceptors and perpetuate pain signaling. This creates a self-reinforcing cycle that amplifies pain and dysfunction [57–59]. Animal studies demonstrate that blocking TNFα reduces inflammation and alleviates pain in radiculopathy, suggesting potential therapeutic strategies [60].

In critical situations, extended nerve compression and inflammation can lead to serious neurological impairments. Conditions such as cauda equina syndrome, characterized by bowel and bladder issues, saddle anesthesia, and weakness in the lower limbs, require immediate surgical action [61]. Similarly, cervical spondylotic myelopathy can result in quadriparesis and walking difficulties, significantly impacting the quality of life [62].

Effective management of these conditions involves addressing both mechanical and inflammatory components. Novel treatments, including biological therapies targeting TNF-α and tailored decompression techniques, show promise in mitigating these complex effects and improving patient outcomes [63].

## 7. Diagnostic Methods

### 7.1 Imaging Modalities

Innovative imaging technologies have significantly changed the assessment of spinal disorders, allowing for unmatched visualization of spinal anatomy and pathologies. MRI is considered the standard for evaluating soft tissues, excelling in defining intervertebral discs, ligaments, the spinal cord, and nerve roots [64]. Recent advancements, such as diffusion weighted imaging (DWI) and contrast-enhanced MRI, offer enhanced insights into microvascular and functional dynamics, facilitating differentiation between neoplastic, infectious, and inflammatory conditions [65]. These techniques are particularly effective for detecting spinal metastases and abscesses with high sensitivity and specificity.

Computed tomography (CT) remains unparalleled for assessing bony structures, enabling accurate detection of fractures, spondylolisthesis, and facet joint degeneration [66]. Innovations like dual-energy CT enhance diagnostic precision by allowing material decomposition to distinguish between bone, soft tissue, and contrast agents, making it valuable for evaluating spinal stenosis and postoperative complications [67]. CT myelography, meanwhile, is a critical alternative for patients contraindicated for MRI, particularly for assessing nerve root impingement [68].

X-rays, despite their limitations in soft tissue detail, continue to be important in initial evaluations by providing quick and cost-effective assessments of surgical misalignment and deformities such as scoliosis and kyphosis [69]. Dynamic radiographs obtained during flexion extension movements are essential for assessing instability in lumbar degenerative spondylolisthesis. These imaging techniques allow for the identification of dynamic instability by evaluating abnormal vertebral motion, providing critical insights into the biomechanical behavior of the spine [70]. Emerging technologies, such as functional MRI and photon-counting CT, promise to further enhance diagnostic capabilities by offering deeper insights into spinal biomechanics and pathology [71]. Together, these imaging modalities enable a comprehensive and tailored approach to managing spinal disorders.

### 7.2 Functional Assessments

Functional assessments are pivotal in the evaluation of spinal disorders, providing objective insights into neuromuscular health. Electrodiagnostic techniques such as electromyography (EMG) and nerve conduction studies (NCS) are essential for the diagnosis and monitoring of conditions that affect nerve and muscle functionality, acting as vital supplements to imaging and clinical examinations [72].

EMG is fundamental to functional diagnostics and involves the placement of thin needles into muscles to monitor electrical activity during both rest and contraction. It reveals abnormal patterns such as fibrillations, fasciculations, or changed motor unit potentials, which signal nerve or muscle dysfunctions. EMG is particularly valuable in diagnosing lumbar radiculopathy by identifying denervation in specific myotomes when clinical findings or imaging results are inconclusive [73]. Abnormal EMG findings are often linked to nerve root compression in the lumbar spine, correlating with conditions like sciatica [74].

Nerve conduction studies, in collaboration with EMG, assesses the speed and strength of electrical impulses traveling through peripheral nerves. By analyzing factors such as conduction velocity and distal latency, NCS can identify conduction blocks, demyelination, or axonal degeneration. NCS has proven effective in detecting conditions like spinal stenosis and peripheral neuropathies [75]. Reduced conduction velocity in the sciatic nerve, for example, frequently signals compression due to herniated lumbar discs [76].

Advancements in electrodiagnostic tools, such as high-density surface EMG and automated NCS systems, have enhanced diagnostic accuracy and efficiency. High-density EMG facilitates detailed, non-invasive motor unit mapping, while automation reduces operator variability, improving reproducibility [77]. However, these tests are not without limitations. Needle-based EMG may cause discomfort, and both EMG and NCS require a high level of operator expertise to ensure accurate interpretation [78]. Results must be integrated with clinical and imaging findings to avoid misdiagnosis.

Despite their limitations, EMG and NCS are crucial tools for the diagnosis and treatment of spinal disorders, providing objective data essential for effective treatment plans. Continued technological improvements are expected to further enhance their precision and comfort for patients.

### 7.3 Emerging Diagnostic Tools

Advancements in diagnostic technologies are transforming the field of spinal care, with new methods such as AI-driven analytics, molecular imaging, and biomarker-based tools providing unparalleled accuracy and personalization. These advancements have the potential to change the way spinal disorders are detected, assessed, and treated, moving towards earlier and more customized interventions.

Artificial intelligence has quickly become a part of diagnostic processes, utilizing algorithms to analyze imaging data with exceptional precision. In the realm of spinal diagnostics, AI-enabled tools evaluate MRI and CT scans to uncover subtle abnormalities that human observers might overlook, including early disc degeneration or minor herniations. Deep learning algorithms enhance diagnostic speed and reliability and facilitate predictions of surgical outcomes, such as adjacent segment disease following spinal fusion [79,80]. These advancements improve clinical decision-making and support personalized treatment strategies.

Molecular imaging techniques like positron emission tomography (PET) and single-photon emission computed tomography (SPECT) provide insights into metabolic and cellular activities within spinal structures. These methods surpass the anatomical focus of traditional imaging by identifying inflammation, infections, and malignancies at the molecular level. For example, fluorodeoxyglucose PET (FDG-PET) has shown high sensitivity in diagnosing vertebral osteomyelitis and spinal metastases, enabling targeted therapies [81, 82]. Hybrid imaging systems such as PET/MRI further enhance diagnostic accuracy by combining functional and structural data, which is particularly useful in assessing spinal tumors and inflammatory conditions [83,84].

Biomarker-based diagnostics represent a promising frontier, focusing on molecular markers like matrix metalloproteinases and pro-inflammatory cytokines. These biomarkers have demonstrated potential in early detection of degenerative processes and chronic pain syndromes, offering a complementary approach to imaging [85,86]. Studies indicate that elevated cytokine levels correlate with early-stage disc degeneration and inflammation, facilitating less invasive diagnostic options [87].

Despite their transformative potential, these technologies face challenges in terms of cost, accessibility, and integration into clinical workflows. AI systems require vast datasets for continued refinement, while molecular imaging and biomarker testing must address standardization and cost-effectiveness issues. Collaborative efforts among researchers, clinicians, and policymakers are essential to overcome these obstacles and ensure equitable implementation of these advanced diagnostics [88,89].

## 8. Treatment Strategies and Adverse Effects

### 8.1 Minimally Invasive Surgeries

**Figure 2:**
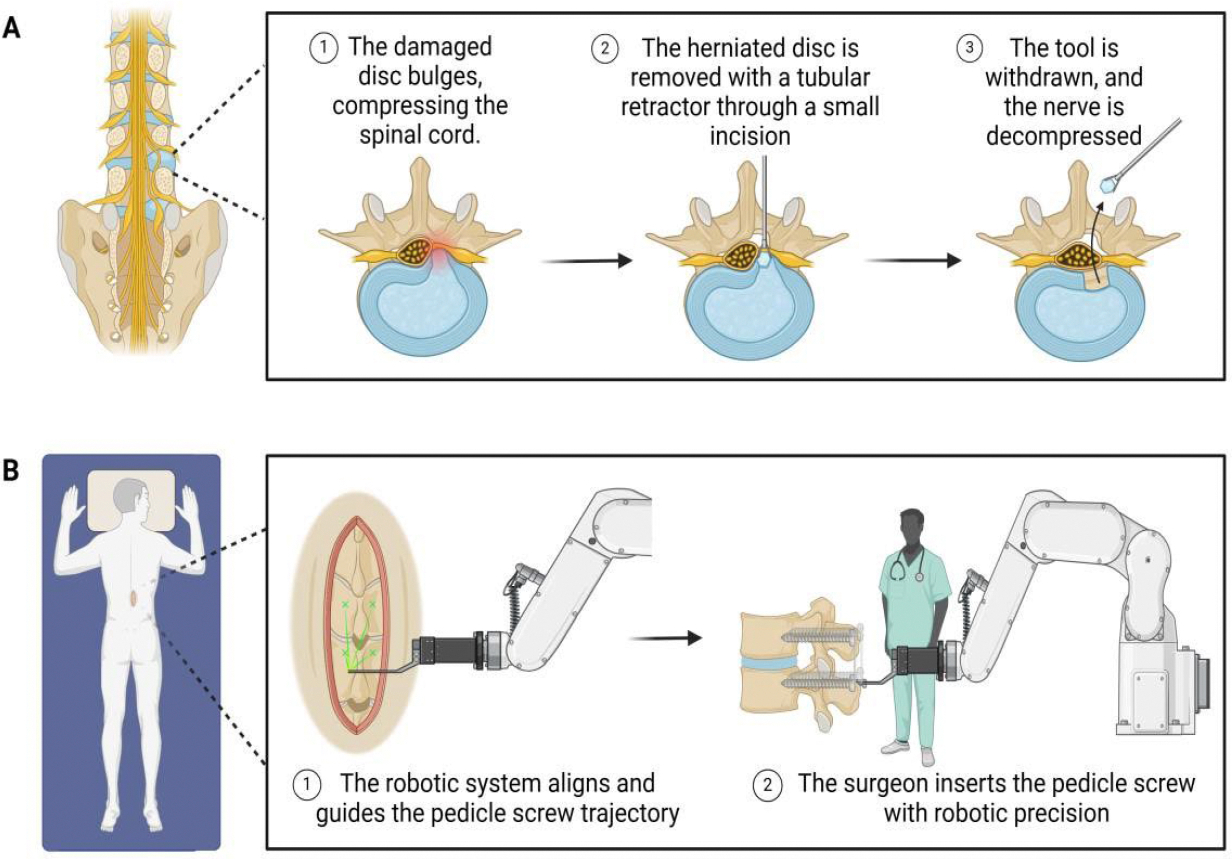
Minimally invasive surgical approaches for spine disorders. (**A**) The process of a minimally invasive endoscopic discectomy is illustrated. (**B**) Robotic-assisted pedicle screw placement is demonstrated.

MIS have transformed the management of spinal disorders, offering a refined alternative to conventional open surgery. Utilizing advanced techniques such as endoscopic discectomy and robotic-assisted spinal fusion as depicted in Figure 2, MIS reduces tissue damage, speeds up recovery, and improves clinical results, signifying a meaningful shift in spinal treatment approaches.

Endoscopic discectomy is a prime example of innovation in MIS, addressing herniated discs and radiculopathy through a tubular endoscope that is inserted through a small incision. This method enables direct visualization and removal of problematic disc material, leading to decreased postoperative pain, shorter hospital stays, and faster recovery times compared to traditional microdiscectomy [90,91]. Technological advancements, including high-definition optics and flexible instruments, have expanded its application across lumbar, thoracic, and cervical disorders [92]. However, its adoption faces challenges, including a steep learning curve and risks such as incomplete decompression or recurrence, emphasizing the importance of experienced practitioners and careful patient selection [93,94].

Robotic-assisted spinal fusion signifies a significant advancement, employing robotic systems for exceptional accuracy in screw placement and alignment during lumbar interbody fusion (Figure 2). The approach reduces risks of improper hardware positioning, lowers blood loss, minimizes infection rates, and promotes faster recovery [95,96]. Studies report improved functional outcomes and patient satisfaction compared to conventional techniques [96]. However, the technology entails high initial costs, longer setup times, and requires surgeon expertise, posing barriers to widespread adoption despite ongoing improvements [22].

While minimally invasive surgery techniques offer substantial benefits, they have limitations. Restricted visualization can increase the risk of incomplete decompression or nerve injury, and sophisticated equipment imposes logistical and financial challenges. Additionally, patients with severe deformities or significant scar tissue may not be ideal candidates for minimally invasive surgery [97]. Nonetheless, ongoing advancements continue to refine these techniques, broadening their applicability and improving patient outcomes.

### 8.2 Complex Deformity Corrections

Surgical correction of complex spinal deformities, such as scoliosis and kyphosis, is a highly intricate yet rewarding field within spinal surgery. These procedures aim to restore spinal alignment, alleviate pain, and enhance functionality. However, they come with significant technical challenges and potential complications, particularly adjacent segment disease.

Scoliosis, characterized by lateral curvature of the spine, is often treated with posterior spinal fusion using instrumentation. Pedicle screw fixation provides robust biomechanical stability, enabling three-dimensional correction of deformities. Modern techniques, such as robotic assisted screw placement and intraoperative imaging systems, have significantly improved precision, reducing complications like screw malposition [98]. Long-term studies validate the efficacy of posterior spinal fusion in halting curve progression and significantly enhancing quality of life for patients with adolescent idiopathic scoliosis, establishing its reliability as a standard treatment [99].

Kyphosis, which is marked by a significant forward curvature of the thoracic spine, typically requires posterior-based osteotomies like pedicle subtraction osteotomy or vertebral column resection. These methods provide considerable sagittal correction but come with considerable risks, including significant blood loss, infections, and neurological deficits [100,101]. Emerging technologies like 3D-printed patient-specific implants are improving surgical outcomes by offering personalized anatomical fit and reducing operative times [102].

Despite their effectiveness, these corrections are associated with a long-term risk of adjacent segment disease, which results from altered spinal biomechanics post-fusion. Adjacent segment disease is a recognized complication of long-segment spinal fusions, with its incidence varying based on patient factors and surgical approaches. Emerging strategies like dynamic stabilization devices and selective fusion techniques aim to reduce this risk, though their long-term efficacy requires further investigation [103,104]. Additionally, technologies like 3D-printed implants and advanced surgical planning software combined with intraoperative navigation are reshaping deformity correction by improving precision and efficiency [105].

### 8.3 Disc Replacement

Artificial disc replacement (ADR) represents a groundbreaking advancement in the treatment of degenerative disc disease and associated spinal conditions, providing a motion sparing option compared to the rigidity seen in spinal fusion [106]. ADR devices utilize materials such as ultra-high molecular weight polyethylene, cobalt-chromium-molybdenum alloys, and titanium alloys to replicate the biomechanical function of natural discs, ensuring flexibility and long-term durability [107]. Clinical studies highlight the efficacy of ADR in both cervical and lumbar regions, showing significant pain relief and functional improvements. Cervical ADR has demonstrated reduced rates of adjacent segment degeneration compared to anterior cervical discectomy and fusion, primarily due to its ability to maintain segmental motion and alleviate biomechanical strain on adjacent levels [108].

Long-term studies have indicated that ADR can significantly reduce the incidence of adjacent segment degeneration compared to traditional fusion techniques [109,110]. Complications associated with ADR, such as implant wear leading to inflammation or osteolysis, and mechanical issues like migration or subsidence, remain significant concerns [111,112]. These risks are heightened in patients with inadequate bone quality or due to improper implantation techniques, occasionally requiring revision surgeries [113,114].

Patient selection is crucial for success. Individuals with advanced facet joint degeneration, osteoporosis, or spinal instability may experience lower success rates, making comprehensive preoperative evaluations essential. Additionally, high costs and inconsistent insurance coverage pose barriers to widespread adoption, particularly in low- and middle-income regions [106].

Innovations aim to address these limitations. The use of advanced biomaterials such as polyether ether ketone (PEEK) and titanium coatings enhances osseointegration and reduces wear, while personalized disc designs tailored to individual biomechanics are showing promise in improving outcomes and reducing complications [115,116]. With ongoing advancements in biomaterials and design, ADR continues to evolve as a cornerstone of modern spinal treatment.

### 8.4 Non-Surgical Approaches

Non-surgical management is a fundamental aspect of treating spinal disorders, especially for patients experiencing mild to moderate symptoms or those who are not candidates for surgery. These methods, which include well established options like physical therapy and medication, as well as newer treatments such as electrical stimulation, focus on alleviating symptoms while being minimally invasive.

Physical therapy is central to conservative treatment, utilizing customized exercise programs and manual techniques to improve strength, flexibility, and posture. Core stabilization exercises reduce stress on the lumbar region and enhance dynamic stability, effectively lessening pain and disability for those with chronic low back pain and degenerative disc disease [117,118]. Supervised therapy consistently outperforms home-based programs, with randomized controlled trials highlighting improved adherence and outcomes in supervised settings [119].

Medication provides symptomatic relief, particularly during acute episodes. Non-steroidal anti-inflammatory drugs (NSAIDs) are a widely used first-line treatment for managing pain and inflammation associated with osteoarthritis and lumbar radiculopathy, particularly for their effectiveness in reducing inflammatory processes and providing analgesia [120]. Muscle relaxants and gabapentinoids are effective for managing spasms and radicular pain, though caution is advised for long-term use due to risks of dependence, particularly with opioids [121].

Electrical stimulation is increasingly recognized as an innovative treatment method. Transcutaneous electrical nerve stimulation (TENS), which uses electrical currents to influence pain signals, demonstrates moderate efficacy in managing chronic spinal pain [122]. Spinal cord stimulation (SCS), involving electrode implantation near the spinal cord, is promising for complex cases like failed back surgery syndrome [123]. Emerging technologies such as percutaneous electrical nerve stimulation (PENS) and pulsed electromagnetic field (PEMF) therapy show potential for reducing pain and promoting healing by targeting underlying inflammation [124].

While generally low risk, the effectiveness of non-surgical treatments can differ based on individual patient characteristics. A comprehensive approach that integrates physical therapy, medication, and innovative methods is essential for maximizing results, with continuous advancements likely to enhance treatment possibilities.

## 9. Outstanding Questions, Gaps in Knowledge, and Challenges

### 9.1 Durability of Innovations

The long-term viability and dependability of new spinal treatments remain significant uncertainties despite their initial potential. ADR, MIS, and biological therapies have shown promising outcomes in the short to medium term. However, their ability to sustain functional benefits and prevent complications over extended periods remains unclear due to the limited duration of follow-up in most studies, which are often confined to the early years after treatment [125,126].

ADR, for example, has become popular as a motion-sparing substitute for spinal fusion. Studies report that while ADR reduces adjacent segment degeneration compared to fusion, lateonset issues like implant failure or loosening often necessitate revision surgeries, which are technically complex and carry higher risks [127]. MIS techniques, such as endoscopic discectomy and robotic-assisted fusion, offer benefits like reduced recovery times, lower morbidity, and enhanced surgical precision. However, concerns remain about their long-term biomechanical impacts, particularly risks of incomplete decompression, altered load distributions, and symptom recurrence, especially in patients with advanced degeneration or deformities [128,129].

Biological therapies, such as platelet-rich plasma (PRP) injections and cell-based treatments, show promise for tissue regeneration but face challenges related to the durability of their effects. Concerns about immune responses and potential tumorigenic risks highlight the need for more extended observation [130,131].

Addressing these challenges requires long-term, multicenter cohort studies with standardized outcome measures and robust data collection. Collaborative registries that monitor real-world patient outcomes can complement clinical trials, providing comprehensive insights into safety and efficacy over decades. Such efforts are critical to establishing the durability and broader applicability of these promising interventions.

### 9.2 Accessibility Issues

Access to advanced spinal technology such as ADR, MIS, and biological therapies is limited by high prices and infrastructure challenges. In areas with limited resources, differences in provider skills, healthcare systems, and insurance coverage worsen these issues. Robotic-assisted surgeries, for instance, demonstrate significant clinical benefits, yet their high initial investment and maintenance expenses restrict availability to affluent institutions [132]. Custom implants and advanced technologies, such as 3D-printed prosthetics, face distribution challenges primarily due to high production costs and limited availability, despite their potential for personalized and effective patient care. The cost of materials and production scalability are significant barriers, hindering widespread adoption [133].

Efforts to mitigate these inequalities include nonprofit collaborations and global partnerships, which have made strides in improving spinal care access in low- and middle-income countries. Subsidized programs for robotic surgeries and portable MIS equipment have shown promise in reducing procedural costs while maintaining quality [134]. Policy measures encouraging local manufacturing and expanding insurance coverage for advanced spinal treatments further enhance accessibility by lowering costs [135].

Innovative models, including telemedicine for pre- and post-operative care and portable diagnostic technologies, are also helping bridge gaps in rural and underserved regions. These solutions not only lower logistical costs but also enable more equitable distribution of high-quality care. However, sustained progress will require large-scale investments, public-private collaborations, and regulatory reforms to integrate advanced spinal technologies into diverse healthcare settings [136].

### 9.3 Research Limitations

Research on spinal conditions encounters various limitations including the scarcity of randomized controlled trials (RCTs), a lack of diversity in study populations, and inadequate economic evaluations. The absence of RCTs, which is the benchmark for medical evaluation, weakens trust in newly developed interventions. Observational designs and small-scale studies are common, introducing biases that restrict generalizability. Treatments like MIS and biological therapies show promise, but their integration into clinical practice is hindered by a lack of direct comparisons with conventional methods [137].

Additionally, geographic and socioeconomic bias further limit the generalizability of results. Research is often disproportionately conducted in affluent areas, leaving out underrepresented groups from low- and middle-income countries or rural areas. Similarly, older adults, women, and ethnic minorities remain underrepresented in spinal research, reducing the relevance of findings to global patient populations [138,139].

Economic analyses are also lacking, particularly for emerging technologies such as robotic systems and 3D-printed implants. Comprehensive cost-effectiveness data are essential for policy decisions, especially in resource-limited settings [19,140].

Efforts to overcome these challenges include conducting multicenter RCTs with standardized outcomes and leveraging global collaborations to increase diversity in research populations. Patient registries and real-world evidence can complement traditional trials, offering insights into long-term efficacy and cost-effectiveness. Expanding funding in underserved regions and implementing equity-focused study designs are vital for improving the inclusivity and applicability of spinal research [135].

## 10. Future Directions in Spinal Care

Progress in spinal care is accelerating rapidly, with breakthroughs in regenerative medicine, technology integration, and individualized strategies transforming the field. These new areas hold the potential to address existing limitations, improve results, and revolutionize the treatment of spinal conditions, though not without challenges.

### 10.1 Regenerative Medicine

Regenerative medicine is poised to revolutionize spinal treatment by leveraging the body’s intrinsic regenerative capabilities through stem cells, biomaterials, and biologics. Mesenchymal stem cells, derived from bone marrow or adipose tissue, exhibit anti-inflammatory and reparative properties, with preclinical studies demonstrating their efficacy in mitigating intervertebral disc degeneration by restoring extracellular matrix and disc height [141]. Initial clinical trials mirror these outcomes, highlighting the potential of mesenchymal stem cells in slowing degeneration progression while improving function [142].

Hydrogels and engineered biomaterials serve as critical platforms in tissue engineering, enabling cell delivery while providing structural support to degenerated discs. These scaffolds enhance disc regeneration and improve biomechanical stability [143]. Furthermore, biologics such as growth factors, specifically, bone morphogenic proteins and platelet-derived growth factor, stimulate reparative cellular processes, offering an adjunctive strategy in tissue restoration [144].

However, significant challenges hinder broad application, including ensuring cell survival, optimizing delivery mechanisms, and managing potential immune responses [145]. Newer innovations, such as injectable biomaterials infused with stem cells and precision medicine strategies, are addressing these limitations by providing personalized approaches tailored to individual biomechanics [146].

Further long-term studies and multicenter trials are essential to validate these advancements’ safety and durability in real-world clinical settings. Collaborative efforts are crucial to overcoming scalability and regulatory barriers, ensuring equitable access to these transformative therapies.

### 10.2 Technological Integration

The integration of robotics, AI, and wearable technologies is revolutionizing spinal care by improving both surgical accuracy and diagnostic precision. Robotic-assisted systems like Major X and Excelsius GPS offer unmatched precision in instrumentation and pedicle screw placement, minimizing intraoperative mistakes and enhancing patient outcomes. Clinical studies report reduced complication rates, decreased intraoperative radiation exposure, and shorter recovery periods compared to conventional freehand techniques [147,148]. However, challenges such as high costs, steep learning curves for surgeons, and substantial infrastructure requirements hinder widespread adoption, particularly in resource-constrained settings [149].

AI continues to revolutionize spinal diagnostics by leveraging machine learning algorithms to detect subtle abnormalities and predict disease progression. For instance, AI models have been shown to improve early detection of intervertebral disc degeneration and predict surgical outcomes for patients undergoing complex spinal fusion surgeries, contributing to personalized treatment plans [150]. AI-driven imaging analysis has also enhanced the identification of conditions like spinal stenosis and adjacent segment disease with higher diagnostic accuracy compared to traditional assessments [151,152].

Wearable technologies complement these advancements by providing real-time data that allow clinicians to dynamically adjust treatment strategies. For example, devices that monitor posture and spinal motion during rehabilitation offer actionable insights, promoting adherence to therapy and improving patient outcomes [153,154]. Innovations such as feedback-enabled wearable sensors for postural correction have shown potential in preventing complications during recovery, particularly after spinal surgeries [155].

Despite these promising developments, adoption barriers persist. The high costs of robotic systems and wearable devices limit access, while the need for rigorous validation studies remains a critical challenge to ensure long-term effectiveness and reliability. Addressing these issues requires collaborative efforts in research, policymaking, and industry investment to promote equitable access to these transformative technologies [156].

### 10.3 Personalized Medicine

Personalized medicine allows customizing treatments based on an individual’s genetic, molecular, and clinical characteristics, which not only enhances therapeutic results but also reduces unnecessary medical procedures. Genetic profiling has become an essential tool, identifying variants linked to scoliosis, degenerative disc disease, and osteoarthritis. Variants that affect extracellular matrix metabolism have been shown to predict susceptibility to intervertebral disc degeneration, enabling preventative care and early intervention strategies [157,158].

Molecular profiling adds another dimension, with biomarkers such as inflammatory cytokines, matrix metalloproteinases (MMPs), and neurofilament light chain serving as indicators of disease progression and response to treatment [159]. Elevated levels of MMPs are associated with the degradation of the extracellular matrix in intervertebral discs, correlating with disease progression. Cytokine profiling has been shown to distinguish inflammatory spinal pathologies from degenerative conditions, aiding in more accurate diagnoses and tailored therapeutic approaches [160,161].

The integration of AI further enhances the possibilities of personalized medicine, as algorithms compile genetic, molecular, and clinical information to improve treatment approaches. AI has been applied to predict outcomes such as adjacent segment disease following spinal fusion, facilitating data-driven surgical decisions. Machine learning tools have also improved the early detection of degenerative disc disease and spinal stenosis, enhancing diagnostic accuracy and treatment planning [162,163].

However, there are significant challenges to implementation. High costs and limited access to genetic and molecular testing pose barriers, especially in low-resource settings. Additionally, ethical concerns regarding data privacy and the potential for genetic discrimination necessitate stringent regulatory frameworks.

## 11. Conclusion

The management of spinal disorders has undergone significant advancements, leveraging technological, surgical, and regenerative breakthroughs. Innovations such as MIS, aADR, regenerative medicine, and integrated technologies like robotics and AI have significantly improved patient outcomes by enhancing precision, reducing recovery times, and offering more personalized care [8,12,15]. MIS techniques, including endoscopic discectomy and robotic assisted spinal fusion, have demonstrated reduced procedural risks and shorter recovery periods, addressing some of the limitations of traditional surgical methods [92,93]. Similarly, regenerative approaches, such as PRP therapies and mesenchymal stem cell applications, show promise for tissue repair and intervertebral disc regeneration [11].

Despite these advancements, challenges persist. High costs and limited accessibility to advanced technologies, particularly in underserved regions, continue to hinder equitable implementation [6,21]. Furthermore, insufficient long-term data on treatments such as biologics and ADR highlight the need for multicenter studies to establish safety and durability over time [42]. These disparities in access, along with the economic burden of spinal disorders globally, emphasize the necessity of addressing systemic barriers to care [3,4].

Looking ahead, integrating regenerative medicine, cutting-edge surgical technologies, and personalized treatment strategies will be crucial to overcoming these challenges. For example, AI and wearable technologies have the potential to revolutionize diagnostics and post-operative care, improving precision and patient engagement [16]. Through collaborative research, policy reforms, and global partnerships, spinal care can continue to evolve, improving outcomes and enhancing the quality of life for patients worldwide.

## 12. Key points:

- Advancements like minimally invasive surgeries, regenerative medicine, and artificial intelligence improve precision, recovery, and patient outcomes.
- Techniques like endoscopic discectomy and robotic-assisted fusion reduce tissue damage, complications, and recovery time.
- Therapies like platelet rich plasma and mesenchymal stem cells show promise in disc regeneration and spinal repair.
- AI improves diagnostic accuracy, while wearable devices aid real-time monitoring and rehabilitation.
- Artificial disc replacement preserves motion better than fusion but risks implant wear and migration.
- High costs, infrastructure needs, and training limit widespread use, especially in low-resource areas.
- Spinal disorders stem from genetics, aging, work hazards, obesity, smoking, and inflammation.
- AI-driven imaging, PET scans, and molecular biomarkers enhance early detection and treatment planning.
- Long-term, large-scale studies are needed to assess safety, cost-effectiveness, and treatment durability.
- Personalized medicine, artificial intelligence, and regenerative therapies will drive innovation and improve accessibility.

## References

[1] Frost BA, Camarero-Espinosa S, Foster EJ. Materials for the spine: Anatomy, problems, and solutions. Materials 12 (2019): 253. 30646556 10.3390/ma12020253 PMC6356370

[2] Chen S, Chen M, Wu X, Global, regional and national burden of low back pain 19902019: A systematic analysis of the Global Burden of Disease study. J Orthop Translat 32 (2021): 49–58. 34934626 10.1016/j.jot.2021.07.005 PMC8639804

[3] Trone MAR, Stover JD, Almarza A, pH: A major player in degenerative intervertebral disks. JOR Spine 7 (2024): e70025. 39703199 10.1002/jsp2.70025 PMC11655178

[4] Alonso-García M, Sarría-Santamera A. The Economic and Social Burden of Low Back Pain in Spain: A National Assessment of the Economic and Social Impact of Low Back Pain in Spain. Spine (Phila Pa 1976) 45 (2020): E1026–E1032. 32706566 10.1097/BRS.0000000000003476

[5] Coates G, Clewes P, Lohan C, Health economic impact of moderate-to-severe chronic pain associated with osteoarthritis in England: a retrospective analysis of linked primary and secondary care data. BMJ Open 13 (2023): e067545. 10.1136/bmjopen-2022-067545 PMC10347464 37438077

[6] Robak N, Broeckelmann E, Mior S, Views and perspectives toward implementing the Global Spine Care Initiative (GSCI) model of care, and related spine care program by the people in Cross Lake, Northern Manitoba, Canada: a qualitative study using the Theoretical Domain Framework (TDF). Implement Sci Commun 5 (2024): 100. 39289772 10.1186/s43058-024-00636-2 PMC11406944

[7] Valenti VE, Vanderlei LCM, Godoy MF. Editorial: Understanding the role of the autonomic nervous system in health and disease. Front Neurosci 18 (2024): 1446832. 38988771 10.3389/fnins.2024.1446832 PMC11233752

[8] Asada T, Simon CZ, Lu AZ, Robot-navigated pedicle screw insertion can reduce intraoperative blood loss and length of hospital stay: analysis of 1,633 patients utilizing propensity score matching. Spine J 24 (2024): 118–124. 37704046 10.1016/j.spinee.2023.09.004

[9] Yang W, Song J, Liang M, Functional Outcomes and New Vertebral Fractures in Percutaneous Vertebroplasty and Conservative Treatment of Acute Symptomatic Osteoporotic Vertebral Compression Fractures. World Neurosurg 131 (2019): e346e352. 31356973 10.1016/j.wneu.2019.07.153

[10] Fan CY, Wu XX, Ji ZW, Application of Enhanced Recovery After Surgery in Patients with Osteoporotic Vertebral Compression Fractures Undergoing Percutaneous Kyphoplasty. World Neurosurg 181 (2024): e339–e345. 37839562 10.1016/j.wneu.2023.10.052

[11] Elmahi OKO, Dedhia M, Dedhia J, Chronic Pain Management: Advance from Pharmaceutical, Technical and Digital Perspective. Genesis J Surg Med 3 (2022): 1–8.

[12] Yamada K, Sudo H, Iwasaki N. Reverse Translational Approach Using Biomaterials and Stem Cells for Intervertebral Disc Degeneration. JMA Journal 7 (2024): 423434. 10.31662/jmaj.2024-0048 PMC11301003 39114621

[13] Supra R, Agrawal DK. Peripheral Nerve Regeneration: Opportunities and Challenges. J Spine Res Surg 5 (2023): 10–18. 36873243 10.26502/fjsrs0052 PMC9983644

[14] Supra R, Wilson DR, Agrawal DK. Therapeutic Potential of “Smart” Exosomes in Peripheral Nerve Regeneration. J Biotechnol Biomed 6 (2023): 189–196. 37388677 10.26502/jbb.2642-91280082 PMC10310314

[15] Zhou S, Yao H, Ma C, Artificial intelligence X-ray measurement technology of anatomical parameters related to lumbosacral stability. Eur J Radiol 146 (2022): 110071. 34864427 10.1016/j.ejrad.2021.110071

[16] Landriel F, Franchi BC, Mosquera C, Artificial Intelligence Assistance for the Measurement of Full Alignment Parameters in Whole-Spine Lateral Radiographs. World Neurosurg 187 (2024): e363–e382. 38649028 10.1016/j.wneu.2024.04.091

[17] Liu J, He X, Gao Z, Design and preliminary biomechanical analysi s of a novel motion preservation device for lumbar spinal disease after vertebral corpectomy. Arch Orthop Trauma Surg 139 (2019): 751–760. 30747259 10.1007/s00402-018-03106-2

[18] Cecchinato R, Bourghli A, Obeid I. Revision surgery of spinal dynamic implants: a literature review and algorithm proposal. Eur Spine J 29 (2020): 57–65. 31916002 10.1007/s00586-019-06282-w

[19] Wilcox B, Mobbs RJ, Wu AM, Systematic review of 3D printing in spinal surgery: the current state of play. J Spine Surg 3 (2017): 433–443. 29057355 10.21037/jss.2017.09.01 PMC5637198

[20] Fiani B, Newhouse A, Cathel A. Implications of 3-dimensional printed spinal implants on the outcomes in spine surgery. J Korean Neurosurg Soc 64 (2021): 659–668. 10.3340/jkns.2020.0272 PMC8273772 34139795

[21] Mehta A, Cheng Ng J, Andrew Awuah W, Embracing robotic surgery in low- and middle-income countries: Potential benefits, challenges, and scope in the future. Ann Med Surg (Lond) 84 (2022): 104803. 36582867 10.1016/j.amsu.2022.104803 PMC9793116

[22] Fiani B, Quadri SA, Farooqui M, Impact of robot-assisted spine surgery on health care quality and neurosurgical economics: A systemic review. Neurosurg Rev 43 (2020): 17–25. 29611081 10.1007/s10143-018-0971-z

[23] Neiva-Sousa M, Carracha C, Nunes da Silva L, Does PlateletRich Plasma Promote Facial Rejuvenation? Revising the Latest Evidence in a Narrative Review. J Cutan Aesthet Surg 16 (2023): 263–269. 38314356 10.4103/JCAS.JCAS_210_22 PMC10833488

[24] Wang SK, Li J, Wang P, Comparison of Four Nutritional Screening Tools for Predicting Postoperative Adverse Events Following Degenerative Spinal Deformity Surgery. Spine (Phila Pa 1976) 49 (2024): 536–546. 38258979 10.1097/BRS.0000000000004933 PMC10962431

[25] Zhao J, Zeng L, Zhao S, Associations of recurrent lumbar disc herniation after percutaneous endoscopic lumbar discectomy with age, body mass index, modic change, disc degeneration and sacral slope: A quantitative review. Exp Ther Med 27 (2024): 195. 38544559 10.3892/etm.2024.12483 PMC10966669

[26] Kuijer PPFM, Van der Molen HF, Visser S. A health-impact assessment of an ergonomic measure to reduce the risk of work-related lower back pain and lumbar disc herniation in construction workers. Int J Environ Res Public Health 20 (2023): 4672. 36901682 10.3390/ijerph20054672 PMC10001867

[27] Wang X, Dong XS, Choi SD, Work-related musculoskeletal disorders among construction workers in the United States from 1992 to 2014. Occup Environ Med 74 (2017): 374–380. 28039200 10.1136/oemed-2016-103943

[28] Liu Z, Cai H, Zhou Z, Causal relationship between basal metabolic rate and intervertebral disc degeneration: a Mendelian randomization study. Eur Spine J 33 (2024): 3352–3358. 38910168 10.1007/s00586-024-08367-7

[29] Zhou J, Mi J, Peng Y, Causal Associations of Obesity With the Intervertebral Degeneration, Low Back Pain, and Sciatica: A Two-Sample Mendelian Randomization Study. Front Endocrinol (Lausanne) 12 (2021): 740200. 34956075 10.3389/fendo.2021.740200 PMC8692291

[30] Altun I, Yuksel KZ. An experimental study on the effects of smoking in the perinatal period and during lactation on the intervertebral disks of newborns. World Neurosurg 102 (2017): 209–216. 27913258 10.1016/j.wneu.2016.11.042

[31] Zhou S, Xu F, Sun Z, Preoperative and follow-up variations of psoas major muscle are related to S1 screw loosening in patients with degenerative lumbar spinal stenosis. BMC Musculoskelet Disord 25 (2024): 418. 38807200 10.1186/s12891-024-07298-0 PMC11134934

[32] Shang Z, Liu Y, Yuan H, Inherited genetic predisposition and imaging concordance in degenerative lumbar scoliosis patients and their descendants. J Orthop Surg Res 19 (2024): 494. 39169360 10.1186/s13018-024-05000-7 PMC11337562

[33] Xue P, Jin H, Zhou X, The role of cytokine receptor-like factor 1 (CRLF1) in facet joint osteoarthritis pathogenesis. Exp Gerontol 195 (2024): 112543. 39128688 10.1016/j.exger.2024.112543

[34] Young JJ, Jensen RK, Hartvigsen J, Prevalence of multimorbid degenerative lumbar spinal stenosis with knee or hip osteoarthritis: a systematic review and meta-analysis. BMC Musculoskelet Disord 23 (2022): 177. 35209884 10.1186/s12891-022-05104-3 PMC8876450

[35] Sundstrup E, Seeberg KGV, Bengtsen E, A Systematic Review of Workplace Interventions to Rehabilitate Musculoskeletal Disorders Among Employees with Physical Demanding Work. J Occup Rehabil 30 (2020): 588–612. 32219688 10.1007/s10926-020-09879-x PMC7716934

[36] Unverzagt S, Bergmann A, Denny K, Physically demanding occupations among females and sex-related differences to develop osteoarthritis of the hip: a systematic review and meta-analysis. J Occup Med Toxicol 19 (2024): 14. 38711071 10.1186/s12995-024-00415-8 PMC11071200

[37] Mauro D, Thomas R, Guggino G, Ankylosing spondylitis: an autoimmune or autoinflammatory disease? Nat Rev Rheumatol 17 (2021): 387404. 10.1038/s41584-021-00625-y 34113018

[38] Uchino Y, Higashi T, Kobayashi N, Risk factors associated with cervical spine lesions in patients with rheumatoid arthritis: an observational study. BMC Musculoskelet Disord 22 (2021): 408. 33941150 10.1186/s12891-021-04285-7 PMC8094562

[39] Reveille JD. Biomarkers for diagnosis, monitoring of progression, and treatment responses in ankylosing spondylitis and axial spondyloarthritis. Clin Rheumatol 34 (2015): 1009–1018. 25939520 10.1007/s10067-015-2949-3 PMC4488904

[40] Shah AA, Schwab JH. Predictive Modeling for Spinal Metastatic Disease. Diagnostics (Basel) 14 (2024): 962. 38732376 10.3390/diagnostics14090962 PMC11083521

[41] Tiwari J, Halder P, Sharma D, Prevalence and association of musculoskeletal disorders with various risk factors among older Indian adults: Insights from a nationally representative survey. PLoS One 19 (2024): e0299415. 39441775 10.1371/journal.pone.0299415 PMC11498719

[42] Rungruangbaiyok C, Vongvaivanichakul P, Lektip C, Prevalence and Associated Factors of Musculoskeletal Disorders among Older Patients Treated at Walailak University Physical Therapy Clinic in Thailand: A Retrospective Study. Int J Environ Res Public Health 21 (2024): 1253. 39338136 10.3390/ijerph21091253 PMC11432095

[43] Fethke NB, Schall MC Jr, Chen H, Biomechanical factors during common agricultural activities: Results of on-farm exposure assessments using direct measurement methods. J Occup Environ Hyg 17 (2020): 85–96. 32069181 10.1080/15459624.2020.1717502 PMC8256625

[44] Qiu Y, Wei X, Tao Y, Causal association of leisure sedentary behavior and cervical spondylosis, sciatica, intervertebral disk disorders, and low back pain: a Mendelian randomization study. Front Public Health 12 (2024): 1284594. 38322127 10.3389/fpubh.2024.1284594 PMC10844448

[45] Guan J, Liu T, Gao G, Associations between lifestyle-related risk factors and back pain: a systematic review and meta-analysis of Mendelian randomization studies. BMC Musculoskelet Disord 25 (2024): 612. 39090551 10.1186/s12891-024-07727-0 PMC11293147

[46] Chanbour H, Chen JW, Ehtesham SA, Time to Surgery in Spinal Trauma: A Meta-Analysis of the World’s Literature Comparing High-Income Countries to Low-Middle Income Countries. World Neurosurg 167 (2022): e268–e282. 35948226 10.1016/j.wneu.2022.07.140

[47] Che YJ, Hou JJ, Guo JB, Low energy extracorporeal shock wave therapy combined with low tension traction can better reshape the microenvironment in degenerated intervertebral disc regeneration and repair. Spine J 21 (2021): 160–177. 32800896 10.1016/j.spinee.2020.08.004

[48] Yazici A, Yerlikaya T. The relationship between the degeneration and asymmetry of the lumbar multifidus and erector spinae muscles in patients with lumbar disc herniation with and without root compression. J Orthop Surg Res 17 (2022): 541. 36514168 10.1186/s13018-022-03444-3 PMC9749279

[49] Zhang AS, Xu A, Ansari K, Lumbar Disc Herniation: Diagnosis and Management. Am J Med 136 (2023): 645–651. 37072094 10.1016/j.amjmed.2023.03.024

[50] Reeves NP, Cholewicki J, van Dieën JH, Are Stability and Instability Relevant Concepts for Back Pain? J Orthop Sports Phys Ther 49 (2019): 415424. 10.2519/jospt.2019.8144 31021689

[51] Vij N, Tolson H, Kiernan H, Pathoanatomy, biomechanics, and treatment of upper cervical ligamentous instability: A literature review. Orthop Rev (Pavia) 14 (2022): 37099. 35936808 10.52965/001c.37099 PMC9353694

[52] Mazurek M, Kulesza B, Gołębiowska N, Factors Predisposing to The Formation of Degenerative Spondylolisthesis-A Narrative Review. Medicina (Kaunas) 59 (2023): 1430. 37629720 10.3390/medicina59081430 PMC10456558

[53] Wang L, Zhang B, Chen S, A Validated Finite Element Analysis of Facet Joint Stress in Degenerative Lumbar Scoliosis. World Neurosurg 95 (2016): 126–133. 27521732 10.1016/j.wneu.2016.07.106

[54] Ma N, Tang X, Li W, Is coronal imbalance in degenerative lumbar scoliosis patients associated with the number of degenerated discs? A retrospective imaging cross sectional study. BMC Musculoskelet Disord 24 (2023): 414. 37231434 10.1186/s12891-023-06558-9 PMC10210366

[55] Awadalla AM, Aljulayfi AS, Alrowaili AR, Management of Lumbar Disc Herniation: A Systematic Review. Cureus 15 (2023): e47908. 38034203 10.7759/cureus.47908 PMC10683841

[56] Verwoerd AJ, Peul WC, Willemsen SP, Diagnostic accuracy of history taking to assess lumbosacral nerve root compression. Spine J 14 (2014): 2028–2037. 24325881 10.1016/j.spinee.2013.11.049

[57] Supra R, Agrawal DK. Mechanobiology of MicroRNAs in Intervertebral Disk Degeneration. J Spine Res Surg 5 (2023): 1–9. 36777190 10.26502/fjsrs0051 PMC9912327

[58] Lyu FJ, Cui H, Pan H, Painful intervertebral disc degeneration and inflammation: from laboratory evidence to clinical interventions. Bone Res 9 (2021): 7. 33514693 10.1038/s41413-020-00125-x PMC7846842

[59] Boakye PA, Tang SJ, Smith PA. Mediators of Neuropathic Pain; Focus on Spinal Microglia, CSF-1, BDNF, CCL21, TNF-α, Wnt Ligands, and Interleukin 1β. Front Pain Res (Lausanne) 2 (2021): 698157. 35295524 10.3389/fpain.2021.698157 PMC8915739

[60] Wang Z, Gu Y, Wang H, FOXG1 interaction with SATB2 promotes autophagy to alleviate neuroinflammation and mechanical abnormal pain in rats with lumbar disc herniation. Ann Med 56 (2024): 2399967. 39624968 10.1080/07853890.2024.2399967 PMC11616759

[61] Benko MJ, Danison AP, Marvin EA, Distal Cauda equina syndrome: A case report of lumbosacral disc pathology and review of literature. Surg Neurol Int 10 (2019): 84. 31528422 10.25259/SNI-152-2019 PMC6744781

[62] Nishimura H, Endo K, Suzuki H, Gait Analysis in Cervical Spondylotic Myelopathy. Asian Spine J 9 (2015): 321–326. 26097646 10.4184/asj.2015.9.3.321 PMC4472579

[63] Xia Y, Wang H, Yang R, Biomaterials delivery strategies to repair degenerated intervertebral discs by regulating the inflammatory microenvironment. Front Immunol 14 (2023): 1051606. 36756124 10.3389/fimmu.2023.1051606 PMC9900107

[64] Vargas MI, Delattre BMA, Boto J, Advanced magnetic resonance imaging (MRI) techniques of the spine and spinal cord in children and adults. Insights Imaging 9 (2018): 549–557. 29858818 10.1007/s13244-018-0626-1 PMC6108966

[65] Mijaljevic MB, Milosevic ZC, Lavrnic SĐ, Assessment of chemical-shift and diffusion-weighted magnetic resonance imaging in differentiating malignant and benign vertebral lesions in oncologic patients: A single institution experience. Radiol Oncol 58 (2024): 527–534. 39361940 10.2478/raon-2024-0049 PMC11604263

[66] Sima S, Chen X, Sheldrick K, Imaging predictors of progression of lumbar spondylolysis to spondylolisthesis: a systematic review. Spine J 24 (2024): 14311442. 10.1016/j.spinee.2024.03.010 38499064

[67] Hamid S, Nasir MU, So A, Clinical Applications of Dual-Energy CT. Korean J Radiol 22 (2021): 970–982. 33856133 10.3348/kjr.2020.0996 PMC8154785

[68] Weisenthal BW, Glassman SD, Mkorombindo T, When does CT myelography add value beyond MRI for lumbar degenerative disease? Spine J 22 (2022): 787–792. 34848342 10.1016/j.spinee.2021.11.016

[69] Ogura Y, Dimar JR, Djurasovic M, Etiology and treatment of cervical kyphosis: state of the art review-a narrative review. J Spine Surg 7 (2021): 422–433. 34734146 10.21037/jss-21-54 PMC8511555

[70] Slikker III W, Orías AAE, Shifflett GD, Image-Based Markers Predict Dynamic Instability in Lumbar Degenerative Spondylolisthesis. Neurospine 17 (2020): 221–227. 32252172 10.14245/ns.1938440.220 PMC7136107

[71] Kuah T, Vellayappan BA, Makmur A, State-of-the-Art Imaging Techniques in Metastatic Spinal Cord Compression. Cancers (Basel) 14 (2022): 3289. 35805059 10.3390/cancers14133289 PMC9265325

[72] Drăghici M, Bădeliță SN, Jercan A, A Comparative Study of the Electroneurographic Findings in Amyloidotic Polyneuropathy in Patients with Light-Chain Amyloidosis and Glu54Gln Transthyretin Amyloidosis. Medicina (Kaunas) 60 (2024): 2027. 39768907 10.3390/medicina60122027 PMC11728403

[73] Yousry S, Elserafy AF, El nisr MM, Role of magnetic resonance neurography in assessment of lumbosacral radiculo-plexopathy: correlation with electrophysiological studies. Egypt J Radiol Nucl Med 54 (2023): 81.

[74] Wang H, Wang Y, Li Y, A diagnostic model of nerve root compression localization in lower lumbar disc herniation based on random forest algorithm and surface electromyography. Front Hum Neurosci 17 (2023): 1176001. 37469999 10.3389/fnhum.2023.1176001 PMC10353737

[75] Chung T, Prasad K, Lloyd TE. Peripheral neuropathy: clinical and electrophysiological considerations. Neuroimaging Clin N Am 24 (2014): 49–65. 24210312 10.1016/j.nic.2013.03.023 PMC4329247

[76] Li W, Liu YC, Zheng CF, Diagnosis of Compressed Nerve Root in Lumbar Disc Herniation Patients by Surface Electromyography. Orthop Surg 10 (2018): 47–55. 29424098 10.1111/os.12362 PMC6594484

[77] Rojas-Martínez M, Serna LY, Jordanic M, High-density surface electromyography signals during isometric contractions of elbow muscles of healthy humans. Sci Data 7 (2020): 397. 33199696 10.1038/s41597-020-00717-6 PMC7670452

[78] Stålberg E, van Dijk H, Falck B, Standards for quantification of EMG and neurography. Clin Neurophysiol 130 (2019): 1688–1729. 31213353 10.1016/j.clinph.2019.05.008

[79] Yagi M, Yamanouchi K, Fujita N, Revolutionizing Spinal Care: Current Applications and Future Directions of Artificial Intelligence and Machine Learning. J Clin Med 12 (2023): 4188. 37445222 10.3390/jcm12134188 PMC10342311

[80] Liawrungrueang W, Park JB, Cholamjiak W, Artificial Intelligence-Assisted MRI Diagnosis in Lumbar Degenerative Disc Disease: A Systematic Review. Global Spine J 15 (2025): 1414105–1418. 10.1177/21925682241274372 PMC11571941 39147730

[81] Lang S, Walter N, Heidemanns S, [18F]FDG PET/CT Imaging Is Associated with Lower In-Hospital Mortality in Patients with Pyogenic Spondylodiscitis-A Registry-Based Analysis of 29,362 Cases. Antibiotics (Basel) 13 (2024): 860. 39335033 10.3390/antibiotics13090860 PMC11429113

[82] Boriani L, Zamparini E, Albrizio M, Spine Infections: The Role of Fluorodeoxyglucose Positron Emission Tomography (FDG PET) in the Context of the Actual Diagnosis Guideline. Curr Med Imaging 18 (2022): 216–230. 34530718 10.2174/1573405617666210916121046 PMC9241079

[83] Courault P, Zimmer L, Lancelot S. Toward Functional PET Imaging of the Spinal Cord. Semin Nucl Med 23 (2024). 10.1053/j.semnuclmed.2024.07.002 39181820

[84] Kogan F, Fan AP, Gold GE. Potential of PET-MRI for imaging of non-oncologic musculoskeletal disease. Quant Imaging Med Surg 6 (2016): 756–771. 28090451 10.21037/qims.2016.12.16 PMC5219958

[85] Tripathi G, Guha L, Kumar H. Seeing the unseen: The role of bioimaging techniques for the diagnostic interventions in intervertebral disc degeneration. Bone Rep 22 (2024): 101784. 39040156 10.1016/j.bonr.2024.101784 PMC11261287

[86] De Simone M, Choucha A, Ciaglia E, Discogenic Low Back Pain: Anatomic and Pathophysiologic Characterization, Clinical Evaluation, Biomarkers, AI, and Treatment Options. J Clin Med 13 (2024): 5915. 39407975 10.3390/jcm13195915 PMC11477864

[87] Xu T, Chen G, Li J, Zhang Y. Exploring causal correlations between inflammatory cytokines and intervertebral disc degeneration: A Mendelian randomization. JOR Spine 7 (2024): e1349. 38993524 10.1002/jsp2.1349 PMC11237178

[88] Malik MMUD, Alqahtani MM, Hadadi I, Molecular Imaging Biomarkers for Early Cancer Detection: A Systematic Review of Emerging Technologies and Clinical Applications. Diagnostics (Basel) 14 (2024): 2459. 39518426 10.3390/diagnostics14212459 PMC11545511

[89] Pinto-Coelho L How Artificial Intelligence Is Shaping Medical Imaging Technology: A Survey of Innovations and Applications. Bioengineering (Basel) 10 (2023): 1435. 38136026 10.3390/bioengineering10121435 PMC10740686

[90] Porto GBF, Cisewski SE, Wolgamott L, Clinical outcomes for patients with lateral lumbar radiculopathy treated by percutaneous endoscopic transforaminal discectomy versus tubular microdiscectomy: A retrospective review. Clin Neurol Neurosurg 208 (2021): 106848. 34339898 10.1016/j.clineuro.2021.106848

[91] Jiang HW, Chen CD, Zhan BS, Unilateral biportal endoscopic discectomy versus percutaneous endoscopic lumbar discectomy in the treatment of lumbar disc herniation: a retrospective study. J Orthop Surg Res 17 (2022): 30. 35033143 10.1186/s13018-022-02929-5 PMC8760683

[92] Ricciardi L, Chaichana KL, Cardia A, The Exoscope in Neurosurgery: An Innovative “Point of View”. A Systematic Review of the Technical, Surgical, and Educational Aspects. World Neurosurg 124 (2019): 136–144. 30660891 10.1016/j.wneu.2018.12.202

[93] Łątka K, Kołodziej W, Pawuś D, Extremely Rare Complications in Uniportal Spinal Endoscopy: A Systematic Review with Unique Case Analyses. J Clin Med 13 (2024): 1765. 38541991 10.3390/jcm13061765 PMC10970759

[94] Lewandrowski KU, Telfeian AE, Hellinger S, Difficulties, Challenges, and the Learning Curve of Avoiding Complications in Lumbar Endoscopic Spine Surgery. Int J Spine Surg 15 (2021): S21–S37. 10.14444/8161 PMC9421222 34974418

[95] Tong M, Zhang S, Zhang W, Efficacy and safety of navigation robot-assisted versus conventional oblique lateral lumbar interbody fusion with internal fixation in the treatment of lumbar degenerative diseases: A retrospective study. Medicine (Baltimore) 103 (2024): e39261. 39121274 10.1097/MD.0000000000039261 PMC11315524

[96] Menta AK, Weber-Levine C, Jiang K, Robotic assisted surgery for the treatment of spinal metastases: A case series. Clin Neurol Neurosurg 243 (2024): 108393. 38917745 10.1016/j.clineuro.2024.108393

[97] Anand N, Mummaneni PV, Uribe JS, Spinal Deformity Complexity Checklist for Minimally Invasive Surgery: Expert Consensus from the Minimally Invasive International Spine Study Group. World Neurosurg 173 (2023): e472–e477. 36841536 10.1016/j.wneu.2023.02.082

[98] Zhang RJ, Zhou LP, Zhang HQ, Rates and risk factors of intrapedicular accuracy and cranial facet joint violation among robot-assisted, fluoroscopyguided percutaneous, and freehand techniques in pedicle screw fixation of thoracolumbar fractures: a comparative cohort study. BMC Surg 22 (2022): 52. 35148749 10.1186/s12893-022-01502-5 PMC8832770

[99] Pishnamaz M, Migliorini F, Blume C, Long-term outcomes of spinal fusion in adolescent idiopathic scoliosis: a literature review. Eur J Med Res 29 (2024): 534. 39497199 10.1186/s40001-024-02052-7 PMC11536752

[100] Nemani VM, Derman PB, Kim HJ. Osteotomies in the Cervical Spine. Asian Spine J 10 (2016): 184–195. 26949476 10.4184/asj.2016.10.1.184 PMC4764533

[101] Mills ES, Mertz K, Faye E, Complication Rates and Utilization Trends of 3Level Posterior Column Osteotomy Compared to Single-Level Pedicle Subtraction Osteotomy. Neurospine 20 (2023): 662–668. 37401085 10.14245/ns.2346222.111 PMC10323336

[102] Wixted CM, Peterson JR, Kadakia RJ, Three-dimensional Printing in Orthopaedic Surgery: Current Applications and Future Developments. J Am Acad Orthop Surg Glob Res Rev 5 (2021): e20.00230–11. 33877073 10.5435/JAAOSGlobal-D-20-00230 PMC8059996

[103] Nouh MR. Spinal fusion-hardware construct: Basic concepts and imaging review. World J Radiol 4 (2012): 193–207. 22761979 10.4329/wjr.v4.i5.193 PMC3386531

[104] Zhao Y, Liang Y, Wang T, A hybrid therapeutic approach for decreasing postoperative complications in patients with adult lumbar degenerative scoliosis. Medicine (Baltimore) 99 (2020): e21221. 32791696 10.1097/MD.0000000000021221 PMC7386975

[105] Rai V, Munazzam SW, Wazir NU, Revolutionizing bone tumor management: cutting-edge breakthroughs in limb-saving treatments. Eur J Orthop Surg Traumatol 34 (2024): 1741–1748. 38461457 10.1007/s00590-024-03876-z

[106] Eskandar T, Ahmed Z, Pan J, The Decline of Lumbar Artificial Disc Replacement. J Spine Res Surg 6 (2024): 86–92. 39267915 10.26502/fjsrs0078 PMC11392031

[107] Pradeep K, Pal B. Biomechanical and clinical studies on lumbar spine fusion surgery: a review. Med Biol Eng Comput 61 (2023): 617–634. 36598676 10.1007/s11517-022-02750-6

[108] Li Z, Liu H, Yang M, A biomechanical analysis of four anterior cervical techniques to treating multilevel cervical spondylotic myelopathy: a finite element study. BMC Musculoskelet Disord 22 (2021): 278. 33722229 10.1186/s12891-021-04150-7 PMC7962321

[109] Xu S, Liang Y, Zhu Z, Adjacent segment degeneration or disease after cervical total disc replacement: a meta-analysis of randomized controlled trials. J Orthop Surg Res 13 (2018): 244. 30285807 10.1186/s13018-018-0940-9 PMC6169069

[110] Deng Y, Li G, Liu H, Mid- to long-term rates of symptomatic adjacent-level disease requiring surgery after cervical total disc replacement compared with anterior cervical discectomy and fusion: a meta-analysis of prospective randomized clinical trials. J Orthop Surg Res 15 (2020): 468. 33046082 10.1186/s13018-020-01957-3 PMC7549243

[111] Werner JH, Rosenberg JH, Keeley KL, Immunobiology of periprosthetic inflammation and pain following ultra-high-molecular-weight-polyethylene wear debris in the lumbar spine. Expert Rev Clin Immunol 14 (2018): 695–706. 30099915 10.1080/1744666X.2018.1511428 PMC6287907

[112] Supra R, Agrawal DK. Innate Immune Response in Orthopedic Implant Failure. J Orthop Sports Med 5 (2023): 9–19. 36777741 10.26502/josm.511500073 PMC9912346

[113] Sukur E, Akman YE, Ozturkmen Y, Particle Disease: A Current Review of the Biological Mechanisms in Periprosthetic Osteolysis After Hip Arthroplasty. Open Orthop J 10 (2016): 241–251. 27499822 10.2174/1874325001610010241 PMC4951796

[114] Kim KR, Chin DK, Kim KS, Revision Surgery for a Failed Artificial Disc. Yonsei Med J 62 (2021): 240–248. 33635014 10.3349/ymj.2021.62.3.240 PMC7934106

[115] Lewandrowski KU, Vira S, Elfar JC, Advancements in Custom 3DPrinted Titanium Interbody Spinal Fusion Cages and Their Relevance in Personalized Spine Care. J Pers Med 14 (2024): 809. 39202002 10.3390/jpm14080809 PMC11355268

[116] Patel NA, O’Bryant S, Rogers CD, Three-Dimensional-Printed Titanium Versus Polyetheretherketone Cages for Lumbar Interbody Fusion: A Systematic Review of Comparative In Vitro, Animal, and Human Studies. Neurospine 20 (2023): 451–463. 37401063 10.14245/ns.2346244.122 PMC10323354

[117] Sethi J, Mohanty U. The impact of core stabilization exercises on pain, dynamic stability, and disability in chronic low back pain patients. J Nat Sci Biol Med 11 (2020): 27–34.

[118] Kuligowski T, Cieślik B, Kuciel N, Effect of Core Stabilizing Training on Young Individuals Presenting Different Stages of Degenerative Disc Disease-Preliminary Report. Int J Environ Res Public Health 18 (2021): 3499. 33800555 10.3390/ijerph18073499 PMC8036822

[119] Frei A, Radtke T, Dalla Lana K, Effectiveness of a Long-term Home-Based Exercise Training Program in Patients With COPD After Pulmonary Rehabilitation: A Multicenter Randomized Controlled Trial. Chest 162 (2022): 1277–1286. 35952766 10.1016/j.chest.2022.07.026

[120] Kikuchi S, Togo K, Ebata N, Database Analysis on the Relationships Between Nonsteroidal Anti-inflammatory Drug Treatment Variables and Incidence of Acute Myocardial Infarction in Japanese Patients with Osteoarthritis and Chronic Low Back Pain. Adv Ther 38 (2021): 1601–1613. 33544304 10.1007/s12325-021-01629-6 PMC7932944

[121] Bhatia A, Engle A, Cohen SP. Current and future pharmacological agents for the treatment of back pain. Expert Opin Pharmacother 21 (2020): 857–861. 32124653 10.1080/14656566.2020.1735353

[122] Johnson MI, Jones G, Paley CA, The clinical efficacy of transcutaneous electrical nerve stimulation (TENS) for acute and chronic pain: a protocol for a meta-analysis of randomised controlled trials (RCTs). BMJ Open 9 (2019): e029999. 10.1136/bmjopen-2019-029999 PMC6830670 31662366

[123] Kapural L, Peterson E, Provenzano DA, Clinical Evidence for Spinal Cord Stimulation for Failed Back Surgery Syndrome (FBSS): Systematic Review. Spine (Phila Pa 1976) 42 (2017): S61–S66. 28441313 10.1097/BRS.0000000000002213

[124] Allen CB, Williamson TK, Norwood SM, Do Electrical Stimulation Devices Reduce Pain and Improve Function? - A Comparative Review. Pain Ther 12 (2023): 1339–1354. 37751060 10.1007/s40122-023-00554-6 PMC10616008

[125] Formica M, Divano S, Cavagnaro L, Lumbar total disc arthroplasty: outdated surgery or here to stay procedure? A systematic review of current literature. J Orthop Traumatol 18 (2017): 197–215. 28685344 10.1007/s10195-017-0462-y PMC5585094

[126] Sangondimath G, Mallepally AR, Marathe N, Degenerative cervical myelopathy: Recent updates and future directions. J Clin Orthop Trauma 11 (2020): 822–829. 32879568 10.1016/j.jcot.2020.07.012 PMC7452218

[127] Skeppholm M, Henriques T, Tullberg T. Higher reoperation rate following cervical disc replacement in a retrospective, long-term comparative study of 715 patients. Eur Spine J 26 (2017): 2434–2440. 28718168 10.1007/s00586-017-5218-0

[128] Pholprajug P, Kotheeranurak V, Liu Y, The Endoscopic Lumbar Interbody Fusion: A Narrative Review, and Future Perspective. Neurospine 20 (2023): 1224–1245. 38171291 10.14245/ns.2346888.444 PMC10762387

[129] Heydar AM, Tanaka M, Prabhu SP, The Impact of Navigation in Lumbar Spine Surgery: A Study of Historical Aspects, Current Techniques, and Future Directions. J Clin Med 13 (2024): 4663. 39200805 10.3390/jcm13164663 PMC11354833

[130] Shimizu Y, Ntege EH, Sunami H, Regenerative medicine strategies for hair growth and regeneration: A narrative review of literature. Regen Ther 21 (2022): 527–539. 36382136 10.1016/j.reth.2022.10.005 PMC9637724

[131] Zhang JY, Xiang XN, Yu X, Mechanisms and applications of the regenerative capacity of platelets-based therapy in knee osteoarthritis. Biomed Pharmacother 178 (2024): 117226. 39079262 10.1016/j.biopha.2024.117226

[132] Lee J, Kim S. Is It the Best Option? Robotic Surgery for Endometriosis. Life (Basel) 14 (2024): 982. 39202724 10.3390/life14080982 PMC11355767

[133] Pathak K, Saikia R, Das A, 3D printing in biomedicine: advancing personalized care through additive manufacturing. Explor Med 4 (2023): 1135–1167.

[134] Bansal E, Kunaprayoon S, Zhang LP. Opportunities for Global Health Diplomacy in Transnational Robotic Telesurgery. AMA J Ethics 25 (2023): E624–E636. 37535507 10.1001/amajethics.2023.624

[135] Rosenkranz B Drug outcomes research and policies - trends and challenges. Front Pharmacol 15 (2024): 1476849. 39253378 10.3389/fphar.2024.1476849 PMC11381405

[136] Totten AM, Womack DM, Griffin JC, Telehealth-guided provider-to-provider communication to improve rural health: A systematic review. J Telemed Telecare 30 (2024): 1209–1229. 36567431 10.1177/1357633X221139892 PMC11389081

[137] Mazzini L, De Marchi F, Buzanska L, Current status and new avenues of stem cell-based preclinical and therapeutic approaches in amyotrophic lateral sclerosis. Expert Opin Biol Ther 24 (2024): 933–954. 39162129 10.1080/14712598.2024.2392307

[138] Pouramin P, Li CS, Busse JW, Delays in hospital admissions in patients with fractures across 18 low-income and middle-income countries (INORMUS): a prospective observational study. Lancet Glob Health 8 (2020): e711–e720. 32353318 10.1016/S2214-109X(20)30067-X PMC10809849

[139] Saran A, White H, Kuper H. PROTOCOL: Effectiveness of interventions for people with disabilities in low- and middle-income countries-an evidence and gap map. Campbell Syst Rev 15 (2019): e1006. 37131467 10.1002/cl2.1006 PMC8356534

[140] Sheha ED, Gandhi SD, Colman MW. 3D printing in spine surgery. Ann Transl Med 7 (2019): S164. 31624730 10.21037/atm.2019.08.88 PMC6778284

[141] Ogasawara S, Schol J, Sakai D, Alginate vs. Hyaluronic Acid as Carriers for Nucleus Pulposus Cells: A Study on Regenerative Outcomes in Disc Degeneration. Cells 13 (2024): 1984. 39682732 10.3390/cells13231984 PMC11639827

[142] Zhang W, Sun T, Li Y, Application of stem cells in the repair of intervertebral disc degeneration. Stem Cell Res Ther 13 (2022): 70. 35148808 10.1186/s13287-022-02745-y PMC8832693

[143] Luo J, Darai A, Pongkulapa T, Injectable bioorthogonal hydrogel (BIOGEL) accelerates tissue regeneration in degenerated intervertebral discs. Bioact Mater 23 (2022): 551562. 10.1016/j.bioactmat.2022.11.017 PMC9764133 36582500

[144] Everts PA, Lana JF, Onishi K, Angiogenesis and Tissue Repair Depend on Platelet Dosing and Bioformulation Strategies Following Orthobiological Platelet-Rich Plasma Procedures: A Narrative Review. Biomedicines 11 (2023): 1922. 37509560 10.3390/biomedicines11071922 PMC10377284

[145] Binch ALA, Fitzgerald JC, Growney EA, Cell-based strategies for IVD repair: clinical progress and translational obstacles. Nat Rev Rheumatol 17 (2021): 158–175. 33526926 10.1038/s41584-020-00568-w

[146] Kim DY, Liu Y, Kim G, Innovative Strategies in 3D Bioprinting for Spinal Cord Injury Repair. Int J Mol Sci 25 (2024): 9592. 39273538 10.3390/ijms25179592 PMC11395085

[147] Gautam D, Vivekanandan S, Mazur MD. Robotic Spine Surgery: Systematic Review of Common Error Types and Best Practices. Oper Neurosurg (Hagerstown). 10.1227/ons.0000000000001293 39037253

[148] Toossi N, Vardiman AB, Benech CA, Factors Affecting the Accuracy of Pedicle Screw Placement in Robot-Assisted Surgery: A Multicenter Study. Spine (Phila Pa 1976) 47 (2022): 1613–1619. 36256605 10.1097/BRS.0000000000004473 PMC9632944

[149] D’Souza M, Gendreau J, Feng A, Robotic-Assisted Spine Surgery: History, Efficacy, Cost, And Future Trends. Robot Surg 6 (2019): 9–23. 31807602 10.2147/RSRR.S190720 PMC6844237

[150] Ahmad HS, Chauhan D, Dagli MM, Machine Learning Models Leveraging Smartphone-Based Patient Mobility Data Can Accurately Predict Functional Outcomes After Spine Surgery. J Clin Med 13 (2024): 6515. 39518657 10.3390/jcm13216515 PMC11547021

[151] Lee S, Jung JY, Mahatthanatrakul A, Artificial Intelligence in Spinal Imaging and Patient Care: A Review of Recent Advances. Neurospine 21 (2024): 474–486. 38955525 10.14245/ns.2448388.194 PMC11224760

[152] Bogdanovic S, Staib M, Schleiniger M, AI-Based Measurement of Lumbar Spinal Stenosis on MRI: External Evaluation of a Fully Automated Model. Invest Radiol 59 (2024): 656–666. 38426719 10.1097/RLI.0000000000001070

[153] Haddas R, Lawlor M, Moghadam E, Spine patient care with wearable medical technology: state-of-the-art, opportunities, and challenges: a systematic review. Spine J 23 (2023): 929–944. 36893918 10.1016/j.spinee.2023.02.020

[154] Rodgers MM, Alon G, Pai VM, Wearable technologies for active living and rehabilitation: Current research challenges and future opportunities. J Rehabil Assist Technol Eng 6 (2019): 2055668319839607. 31245033 10.1177/2055668319839607 PMC6582279

[155] Owlia M, Kamachi M, Dutta T. Reducing lumbar spine flexion using real-time biofeedback during patient handling tasks. Work 66 (2020): 41–51. 32417812 10.3233/WOR-203149 PMC7369082

[156] Thacharodi A, Singh P, Meenatchi R, Revolutionizing healthcare and medicine: The impact of modern technologies for a healthier future-A comprehensive review. Health Care Sci 3 (2024): 329–349. 39479277 10.1002/hcs2.115 PMC11520245

[157] Cherif H, Mannarino M, Pacis AS, Single-Cell RNA-Seq Analysis of Cells from Degenerating and Non-Degenerating Intervertebral Discs from the Same Individual Reveals New Biomarkers for Intervertebral Disc Degeneration. Int J Mol Sci 23 (2022): 3993. 35409356 10.3390/ijms23073993 PMC8999935

[158] Rajasekaran S, Tangavel C, Soundararajan DCR, Proteomic Signatures of Healthy Intervertebral Discs From Organ Donors: A Comparison With Previous Studies on Discs From Scoliosis, Animals, and Trauma. Neurospine 17 (2020): 426–442. 32615701 10.14245/ns.2040056.028 PMC7338947

[159] Shnayder NA, Ashhotov AV, Trefilova VV, Cytokine Imbalance as a Biomarker of Intervertebral Disk Degeneration. Int J Mol Sci 24 (2023): 2360. 36768679 10.3390/ijms24032360 PMC9917299

[160] Altun I Cytokine profile in degenerated painful intervertebral disc: variability with respect to duration of symptoms and type of disease. Spine J 16 (2016): 857–861. 26975459 10.1016/j.spinee.2016.03.019

[161] Maksymowych WP. Biomarkers for Diagnosis of Axial Spondyloarthritis, Disease Activity, Prognosis, and Prediction of Response to Therapy. Front Immunol 10 (2019): 305. 30899255 10.3389/fimmu.2019.00305 PMC6416369

[162] Galbusera F, Casaroli G, Bassani T. Artificial intelligence and machine learning in spine research. JOR Spine 2 (2019): e1044. 31463458 10.1002/jsp2.1044 PMC6686793

[163] Hornung AL, Hornung CM, Mallow GM, Artificial intelligence in spine care: current applications and future utility. Eur Spine J 31 (2022): 2057–2081. 35347425 10.1007/s00586-022-07176-0

